# The ALR-RSI score can be used to evaluate psychological readiness to return to sport after acute Achilles tendon tear

**DOI:** 10.1007/s00167-023-07548-z

**Published:** 2023-08-23

**Authors:** E. Shitrit, E. Valentin, N. Baudrier, Y. Bohu, G. Rauline, R. Lopes, T. Bauer, A. Hardy

**Affiliations:** 1 Clinique Du Sport, 28 Boulevard Saint-Marcel, 75005 Paris, France; 2 Clinique Jouvenet, 6 Sq. Jouvenet, 75016 Paris, France; 3 34 Rue Michal, 75013 Paris, France; 4 chirurgie orthopedique, Pied cheville Nantes Atlantique, sante Atlantique, avenue Claude-Bernard, 44800 Saint-Herblain, France; 5 Clinique Brétéché, 3, rue de la Béraudière, BP 54613, 44046 Nantes Cedex 1, France; 6 Service de chirurgie orthopedique et traumatologique, hopital Ambroise Paré, universite Paris-Saclay, Assistance publique-Hôpitaux de Paris, 9, avenue Charles-de-Gaulle, 92100 Boulogne-Billancourt, France

**Keywords:** Achilles tendon tear, Achilles repair, ALR-RSI, Return to sport, Psychological

## Abstract

**Purpose:**

The return to sport is one of the main goals following Achilles tendon tear repair. Several psychological factors influence the return to sport after a sports injury. The traditional tools to assess the return to sport do not take into account psychological factors. The ankle ligament reconstruction-return to sport injury (ALR-RSI), validated for ankle instability, is a score to evaluate psychological readiness to return to sport. The goal of this study was to validate the ALR-RSI score for the assessment of the readiness to return to sport after Achilles tendon repair.

**Methods:**

The ALR-RSI score, adapted from the anterior cruciate ligament-return to sport injury (ACL-RSI) score used following knee ligament reconstruction, was validated according to the international COSMIN methodology. Patients operated for Achilles tendon repair responded to the questionnaire during the rehabilitation period. The EFAS, FAAM and VISA-A scores were used as reference questionnaires.

**Results:**

A total of 50 patients were included. The ALR-RSI score was strongly (*r* > 0.5) correlated to the EFAS score: *r* = 0.68 [0.50–0.80] the FAMM sport score: r = 0.7 [0.52–0.84] the FAAM AVQ score (*r* = 0.6 [0.35–0.78]), and the VISA-A score (*r* = 0.54 [0.26–0.76]). The discriminant validity was good with the ALR-RSI, which was significantly lower in the patients that did not return to sport: 60.7 (40–81.4) compared to those that did: 83.2 (64.3–100) *p* = 0.001. Reproducibility was excellent with an intra-class correlation coefficient ρ of 0.99 [097–1.00]. The internal consistency was excellent (alpha coefficient = 0.95).

**Conclusion:**

The ALR-RSI score provides a valid, reproducible assessment of the psychological readiness to return to sport in patients who undergo surgical Achilles tendon suture repair.

**Level of evidence:**

III.

## Introduction

The Achilles tendon is the largest, most powerful tendon in the human body. Achilles tendon tears mainly occur in middle-aged patients during sports. The number and the incidence of Achilles tendon tears have increased in the past decades [14]. The therapeutic options include conservative, functional treatment and surgical repair. Although there is no consensus on the optimal management of acute Achilles tendon ruptures, surgical repair seems to decrease the risk of re-tear and is the most reliable therapeutic option in a young and active population [11, 13, 24].

The return to sport is one of the main considerations in this population. Although the results of surgical repair of the Achilles tendon are good, recent studies in the literature report that 20 to 25% of patients cannot return to sport [15, 35]. Although physical factors such as muscular weakness [22] or reduced endurance [5] can explain the failure to return to sport, several psychological factors seem to influence recovery during the post-injury period (including the rehabilitation period and the re-athletization phase) and play a major role in this process [10].

Clement et al. [8] analysed the psychosocial responses of athletes who suffered an injury that restricted their sport participation (ACL reconstruction, fractures, rotator cuff repair, and elbow lesion). According to the authors [8], there are three phases to the period of rehabilitation. During the acute post-injury period, athletes express negative emotions that are correlated to the severity of the injuries and the supposed delay before the return to sport. During the reeducation phase, the cognitive reactions are ambivalent, and frustration is the main emotion. During the return to sport phase, athletes express doubt about their capacity to return to sport at the desired level of play, become nervous and anxious and are afraid of injuring themselves again [8].

The traditional tools to assess the return to sport do not take into account psychological factors. Webster et al. [33] developed a questionnaire with 12 items (anterior cruciate ligament-return to sport injury (ACL-RSI)) to evaluate the psychological readiness before the return to sport following anterior cruciate ligament reconstructions. This score has been adapted to other sports-related diseases: shoulder instability (SIRSI) [12], femoroacetabular impingement (Hip-RSI) [34], and ankle instability (ankle ligament reconstruction-return to sport injury ALR-RSI) [2, 26, 29]. There is no tool, to date, to analyse the psychological readiness to return to sport after Achilles tendon repair.

To our knowledge, this is the first study that evaluates a score taking into account psychological factors, after repair of the Achilles tendon.

The main goal of this study was to validate the ankle ligament reconstruction-return to sport injury (ALR-RSI) score to evaluate the psychological readiness to return to sport following an Achilles tendon tear.

The hypothesis was that the ALR-RSI score could discriminate the readiness to return to sport in patients operated for an Achilles tendon tear.

## Materials and methods

This study was approved by the ethics committee (COS-RGDS-2022-10-003-HARDY-A).

### Patient characteristics

All patients who engaged in sports activities, at least once a month, and who underwent surgery for Achilles tendon repair in three surgical units between April 2021 and April 2022 were included. The exclusion criteria were: age under 18 years old, patients who did not practise a sport before the injury, treated by mini-open of percutaneous procedures.

Open surgical repair of a recent Achilles tendon tear was performed in all patients of different centres. Patients were immobilised in a boot-with-heel lifts and no weight-bearing for 3 weeks, then, in same position with weight-bearing allowed. Afterwards, they followed an equivalent rehabilitation protocol, including joint range of motion recovery and muscle strengthening. Fifty patients were included, 8 women and 42 men. Regarding the level of sports activity of the patients: Five patients (10%) were professional athletes (1 basket, 2 handball, 2 football), 20 patients (40%) practised a sport at the competitive level, and 25 (50%) at the amateur level. The distribution of different types of sports is detailed in the table (Table 1).

**Table 1:** Baseline characteristics

Parameters		Values	*N*	
Gender		Female	8	16%
		Male	42	84%
Age			50	36.5 (± 10.7)
Follow-up (months)			50	6.8 (6; 9)
ALR-RSI total			50	65.6 (3.3; 98.3)
VISA total			50	77.3 (35.0; 100.0)
FAAM Sport			50	75.1 (31.3; 100.0)
FAAM AVQ			50	93.4 (28.6; 100.0)
EFAS total			50	76.7 (27.5; 100.0)
Sport recovery		Yes	11	22%
		No	39	78%
If yes		Inferior level	7	63.6%
		Same level	4	36.4%
Sport level		Casual leisure level	3	6%
		Regular leisure level	22	44%
		Competitive	20	40%
		Professional	5	10%
Sport	With impulsion	Running	5	10%
		Tennis	6	12%
		Basketball	7	14%
		Football	16	32%
		Handball	6	12%
		Volley	2	4%
		Others	6	12%
	Without impulsion	Walking	1	2%
		Ski	1	2%

#### The ALR-RSI score

**Fig. 1:**
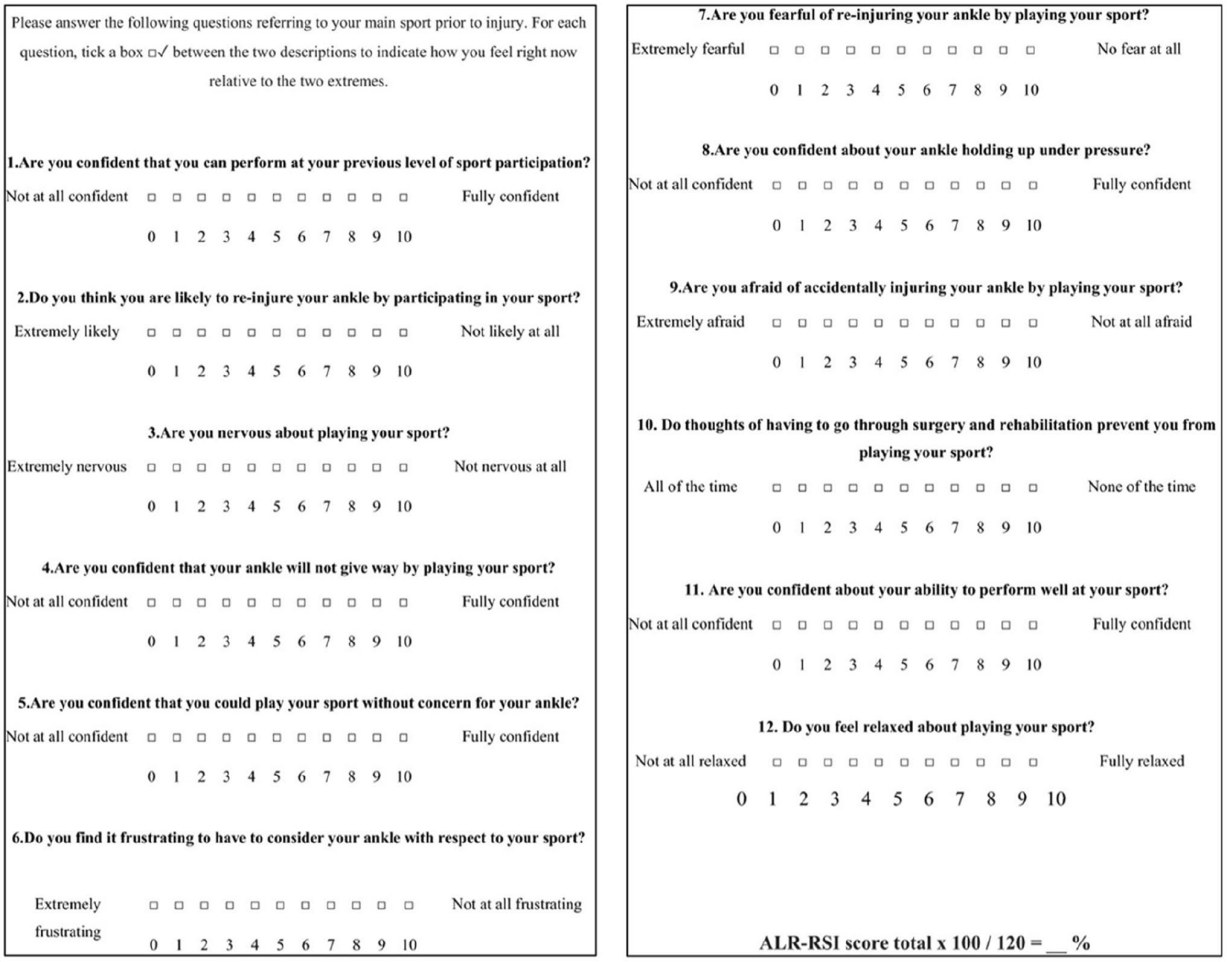
The English version of ALR-RSI score

The French version of ALR-RSI was adapted and validated in a French speaking population [2, 4]. The questionnaire has substituted the word “knee” for “ankle” in questions 2, 4, 5, 6, 7, 8, and 9. For example, question 2 “Do you think you are likely to re-injure your knee by participating in your sport?” was altered to “Do you think you are likely to re-injure your ankle by participating in your sport?” (Fig. 1).

The score was validated in ankle instability injuries, whether treated by repair [26] or arthroscopic ligament reconstruction [29]. There were no modifications in the use of the ALR-RSI score for Achilles rupture compared to the one used ankle instability.

This score is based on 3 psychological factors correlated to the return to sport: emotions, such as fear, confidence and self-esteem [27].

Factors influencing the return to sport such as the type or level of sport, have been searched.

#### Validity and reproducibility of the ALR-RSI

The reference scores used were the Victorian Institute of Sport Assessment (VISA-A) [9], the European Foot and Ankle Society (EFAS) [28] and the Foot Ankle Ability Measurement (FAAM) [21] scores.

The patients were seen between 6 and 9 months after surgery in consultation with a physical therapist, and completed the ALR-RSI, VISA-A, EFAS, and FAAM scores as well as questions concerning the return to sport: Did you resume the sport you were practising before the injury? Yes/No. If yes, at which level? Same level/Inferior level. In order to evaluate reproducibility of ALR-RSI score, a test/retest has been performed in 10 patients chosen randomly at 15-day intervals.

#### Statistical methods

In this study, a sample size of 50 participants resulted in a two-sided 95% confidence interval with a width < 0.33 when the estimated Spearman’s rank correlation is > 0.70. The number of events and their percentages were used to describe qualitative variables, whilst means, minima and maxima were was used to describe quantitative variables unless mentioned otherwise. All questionnaire scores were reported out of 100, with 0 being very poor and 100 excellent. The Spearman correlation coefficient was used to estimate the correlation between the ALR-RSI, the EFAS, the VISA and the FAAM scores. This coefficient was considered to be “weak” *r* < 0.3, “moderate” 0.3 < *r* < 0.5 or “strong” *r* > 0.5. The discriminant validity of the ALR-RSI between the group of patients that “returned to sport” and the group that “did not return to sport” was tested by the Wilcoxon test. The validity of the ALR-RSI and the other questionnaires to determine the readiness to return to sport was evaluated and assessed by receiver operating characteristic (ROC) curve statistics. In general, an AUC of 0.5 suggests no discrimination, 0.7–0.8 is considered acceptable, 0.8–0.9 is considered excellent, and more than 0.9 is considered to be outstanding. The feasibility was estimated by the missing responses, as well as the ceiling and floor effects. To determine internal consistency, the Cronbach alpha was calculated and the correlation between the 12 items of the ALR-RSI was considered to be “excellent” if *α* > 0.90. The reliability was evaluated by the *ρ* intra-class correlation coefficient (ICC). The reproducibility was “excellent” if *ρ* ≥ 0.75, “good” if 0.40 < *ρ* < 0. 75 and “weak” if *ρ* ≤ 0.40. A *p* value < 0.05 was considered to be statistically significant. All statistical analyses were performed using R software (version 4.2).

## Results

### Return to sport

Eleven of the 50 patients (22%) had returned to sport at 6.8 months of follow-up. Four (36.4%) of these patients returned to sport at the preinjury level of play and 7 patients (63.6%) at a lower level of play (Table 1).

Amongst the patients who resumed sports, one patient was competing in football and was able to return to the field after 9 months. The others were regular leisure level athletes who practised running for four patients, basketball for two, handball for one, tennis for one, fitness for one, walking for one (Table 1).

No correlation was found between the type, level of sport, and the return to sport (Table 2).

**Table 2:** Correlation between level and type of sport, and return to sport

	Return to sport		*P*-value
	Yes	No	
Level of sport			n.s
Competitive or professional	3 (12%)	22 (88%)	
Regular or occasional	8 (32%)	17 (68%)	
Type of sport			n.s
With impulsion	9 (20%)	36 (80%)	
Without impulsion	1 (50%)	1 (50%)	

### Construct validity

The ALR-RSI was strongly (*r* > 0.5) and significantly correlated to the EFAS: *r* = 0.68 [0.50–0.80] and FAMM sport scores: *r* = 0.7 [0.52–0.84] as well as to the FAAM AVQ (*r* = 0.6 [0.35–0.78]), and VISA-A (*r* = 0.54 [0.26–0.76]) scores. (Table 3).

**Table 3:** Correlation between the ALR-RSI and the other questionnaires

Coefficient	ALR-RSI	EFAS/100	FAAM sport	FAAM AVQ	VISA
	65.6 (22.2)	76.7 (16.6)	75.1 (18.7)	93.4 (11.5)	77.3 (18.2)
Spearman		0.68 [0.50–0.80]	0.70 [0.52–0.84]	0.60 [0.35–0.78]	0.54 [0.26–0.76]

### Discriminant validity

The ALR-RSI score was significantly lower in the population of patients that did not return to sport: 60.7 (40–81.4), compared those who returned to sport: 83.2 (64.3–100) *p* = 0.001.

A Youden index of 0.62 was observed for a cut-off score of 77.5 points corresponding to a sensitivity of 82% and a specificity of 79%.

**Fig. 2:**
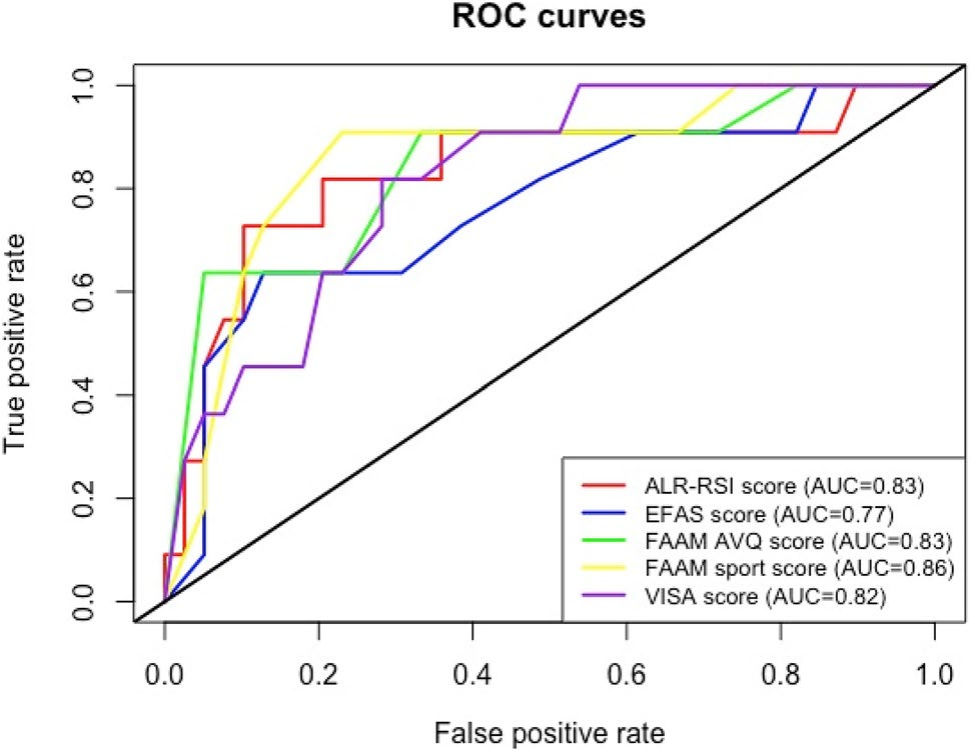
Receiver operating characteristic (ROC) curves for ALR-RSI, VISA, EFAS and FAAM to identify patients who returned to sport. None of the AUC (Area Under the Curve) were statistically different

The ALR-RSI score had a high discriminant capacity (AUC = 0.83) that was comparable to the other scores: EFAS (AUC = 0.77), FAAM AVQ (AUC = 0.83), FAAM sport (AUC = 0.86), VISA-A (AUC = 0.82) (Fig. 2).

### Feasibility

All the participants responded to all the questions.

The floor effect, corresponding to the percentage of patients with the lowest score for each questions, was 0%, and the ceiling effect, corresponding to the percentage of patients with the highest score for each question varied between 0 and 10% depending on the questions, but none of the patients presented with a complete score of 100%.

### Reliability

The reliability was evaluated by the intra-class correlation coefficient ρ (ICC). In the present study, the reproducibility was «excellent» with an intra-class correlation coefficient ρ of 0.99 [0.97–1.00]. During the first test, the median ALR-RSI score was 56.5 (32.5–83.3), and 57.1 (33.3–83.3) for the retest (Table 4).

**Table 4:** Reproducibility of the ALR-RSI score with the test–retest

Coefficient	ALR-RSI 1 (/100)	ALR-RSI 2 (/100)
	56.5 ( 32.5; 83.3)	57.1 (33.3; 83.3)
ICC		*ρ* = 0.99 [0.97–1.00]

### Internal consistency

The Cronbach alpha coefficient was 0.95, which signifies that the internal consistency of the scale measuring the strength of the correlation between the 12 items was «excellent».

## Discussion

The main result of this study was that the ALR-RSI score is a valid and reproducible score to evaluate psychological readiness to return to sport after an Achilles tendon tear.

In our series, sport was resumed by 22% of the patients after an average of 6.8 months. A systematic review in the literature reported that 80% of patients were capable of returning to sport 6 months after an Achilles tendon tear [35]. However, large variations amongst the included studies were observed. The rate of return to sport varied between 18.6 and 100% according to the studies. However, in most studies, the postoperative follow-up was 2 years [35], which explains the higher rate of return to sport than in our series. In the literature, the delay to the return to sport varies between 9 and 12 months depending on the studies [6, 17, 23].

In this study, 6- and 9-month follow-ups were chosen to improve the discriminant capacity of ALR-RSI score by attempting to obtain a similar number of patients who have returned to sport and patients who have not returned to sport. However, a 12-month follow-up could have been more appropriate to improve discriminant capacity.

Trofa et al. [32] reported results in a population of 62 athletes (basketball, baseball and US football) at 1 year and 2 years of follow-up. In their series, 30.6% of the players did not return to high level sports. When athletes returned to sport, their playing time was considerably reduced and their athletic performance was significantly poorer than before the injury [32].

In our study, no correlation was found between the type and level of sports and the return to sports. This observation could be explained by a lack of power due to the small number of patients.

ALR-RSI score was initially developed to assess the return to sports after ankle instability surgery. Some factors are common to instability and Achilles rupture, such as the fear of re-injury and confidence in the ankle. However, some factors are specific to Achilles rupture, notably the loss of propulsion that can impact the return to sports, especially sports requiring jumps.

Numerous scores exist to evaluate Achilles tendon repair. The EFAS, VISA-A, and FAAM scores were used because they are the most frequent scores in the literature. [1, 7, 18, 20, 31]. Nevertheless, these scores only evaluate physical recovery and do not take into account the psychological aspects of the return to sport. Indeed, during postoperative rehabilitation, patients describe a gradual decrease in negative emotions linked to the initial injury (depression, anger, and anxiety) and progress to positive emotions, such as confidence and the feeling of being ready to return to sports [3]. Ardern et al. [3] identified the factors influencing the return to sport such as motivation, self-esteem, and confidence. One of the main negative emotions is fear which may have several aspects. Athletes can be afraid to reproduce the movement that caused the original injury. In these cases, a discussion can be helpful so that athletes can express their apprehension or fears and refocus their attention on the positive aspects of competing [25]. Thus, the ALR-RSI can be used to evaluate the patient’s feelings and help guide them in the return to sport.

Recently, Slagers et al. [30] evaluated associations between psychological factors during rehabilitation and functional outcomes 12 months after Achilles tendon rupture. They showed that psychological readiness and confidence to return to sport increased over time and kinesiophobia decreased over time whilst motivation levels remained high [30].

Kvist et al. [19] have suggested guidelines to improve the management of athletes after an injury. Athletes primarily fear new injuries or the recurrence of previous ones, enduring lifelong pain, inability to return to their sport at the same level, and the overall impact on their lives. It is crucial for athletes to have time to make informed decisions regarding treatment, career choices, and risk assessment for future pain and disability. Whilst quick decisions and interventions are crucial for professional athletes, most non-professionals should focus on tissue healing and realistic recovery timeframes [19]. Accessing accurate information about injuries, treatment options, and recovery requirements is vital. Some athletes may choose to quit sports to minimise the risk of new injuries, whilst others may opt for less risky activities. For those who decide to return to their sport, efforts must be made to alleviate the fear of re-injury as fear itself can increase the risk and hinder rehabilitation progress. Inability to return to sports negatively impacts long-term quality of life, and psychological interventions, physical rehabilitation, and support from coaches, friends, therapists, and doctors play essential roles in reducing fear and promoting recovery [19].

The discriminant capacity of the ALR-RSI score was proven. The score was much lower in the population that did not return to sport. Thus, it is an effective tool to analyse the return to sport after an Achilles tendon tear. The discriminant validity of the VISA-A score in Achilles tendinopathies was evaluated by Kaux et al. [16]. The score was evaluated in different categories of the population: healthy, pathological and at risk. The authors found a weak and moderate correlation and concluded that the discriminant validity of the VISA-A score needed to be confirmed [16]. The FAAM score was shown to have good discriminant validity [21].

Compared to open questioning, the use of a quantitative score provides a more reproducible and standardised evaluation of the return to sport. The ALR-RSI score seems to be an interesting tool in daily practice because the patient’s progress can be evaluated. Moreover, patients with potential difficulties can be identified and psychological support can be provided to optimise the return to sport.

ALR-RSI score was initially developed to assess the return to sports after ankle instability surgery. Some factors are common to instability and Achilles rupture, such as the fear of re-injury and confidence in the ankle. However, some factors are specific to Achilles rupture, notably the loss of propulsion that can impact the return to sports, especially sports requiring jumps.

Nevertheless, this study has certain limitations, in particular the small patient population. Furthermore, the ATRS (Achilles Tendon Total Rupture) score could have been used, which specifically evaluates patients with Achilles tendon rupture.

## Conclusion

The results of this series show that the ALR-RSI score is a valuable tool to assess the psychological readiness to return to sport in patients who undergo surgical Achilles tendon suture repair.

## Abbreviations

ALR-RSI: Ankle ligament reconstruction-return to sport injury
ACL-RSI: Anterior cruciate ligament-return to sport injury
VISA-A: Victorian institute of sport assessment-Achilles
EFAS: European Foot and Ankle Society
FAAM: Foot ankle ability measurement
ROC: Receiver operating characteristic
AUC: Area under the curve
